# Multilevel analysis of intimate partner violence and associated factors among reproductive-age women: Kenya demographic and health survey 2022 data

**DOI:** 10.1186/s12889-024-19012-9

**Published:** 2024-06-01

**Authors:** Mamaru Melkam, Setegn Fentahun, Gidey Rtbey, Fantahun Andualem, Girum Nakie, Techilo Tinsae, Mulat Awoke Kassa, Bezawit Melak Fente

**Affiliations:** 1 College of Medicine and Health Science, Department of Psychiatry, University of Gondar, Gondar, Ethiopia; 2 College Health Science, Departments of Psychiatry, Woldiya University, Woldiya, Ethiopia; 3 College of Medicine Health Science, School of Midwifery, Department of General Midwifery, University of Gondar, Gondar, Ethiopia

**Keywords:** Intimate partner violence, Demographic, Health survey, Kenya

## Abstract

**Introduction:**

Intimate partner violence is a human rights violation that often involves violence against women, which appears to be the most prevalent type of abuse. Intimate partner violence is a major global public health issue that includes physical, emotional, and sexual violence. The prevalence of intimate partner violence in Africa is high. The burden of intimate partner violence among reproductive-age women is high in Kenya. Therefore, the main aim of this study is to determine the associated factors of intimate partner violence among reproductive-age women at the individual and community level from the recent Demographic and Health Survey (DHS) 2022 data of Kenya.

**Methods:**

The Kenya National Demographic and Health Survey data of 2022 was used for this study. The overall sample size for this study was 14,612, which focused on women aged 15 to 49 years who had ever been partnered and responded to the domestic violence module. Multilevel logistic regression models to determine the prevalence and associated factors at the individual and community level with intimate partner violence with a 95% Confidence Interval (CI) and Adjusted Odds Ratio (AOR).

**Result:**

The overall prevalence of intimate partner violence was 41.1% with a 95% CI (40.07%, 42.60. Male-headed households, poorest and middle wealth status, partner alcohol use, separated/widowed current marital status, and low education of women were statistically significantly associated with intimate partner violence at the individual level variables in this study.

**Conclusions:**

The prevalence of intimate partner violence was high. Educating women, reducing partner alcohol use, and improving the economic status of women, were crucial in mitigating the burden of intimate partner violence. The intimate partners are supposed to respect the rights of women.

## Introduction

The World Health Organization (WHO) defines Intimate Partner Violence (IPV) as the deliberate act of an intimate partner or former spouse that results in sexual assault, physical assault, emotional harm, and/or economic violence [1, 2]. IPV is the deliberate act of an intimate partner or former spouse that results in sexual assault, physical assault, economic assault, and emotional harm. Due to serious human rights violations, physical violence against women is becoming more widely acknowledged [3]. Different studies indicate that women in developing countries experience a higher rate of intimate partner violence than women from developed countries [1, 3]. Women’s intimate partner violence is impacted by physical violence both directly, as in the case of injuries, and indirectly by stress from its continuous effect of assault. The impact on women’s health increases with the severity of physical abuse, and these effects seem to compound over time [4]. It can lead to relationship discontent or conflict, male domination in the home, unstable economic conditions, and high rates of general violence in society [5].

Adolescents and young adults who experience IPV at this age are more likely to have long-term effects on their health, emotional, and financial well-being [6]. Laws related to the protection of gender and human rights have long been affected by violations of women’s rights in this context. These challenges are compounded by the multi-sectoral nature of these policies, which further complicates their execution [3, 6]. Intimate partner violence (IPV) can have several negative repercussions on one’s physical, emotional, sexual, and reproductive health, in addition to injuries. Sexual violence can lead to decreasing efficiency at work, this type of violence raises the possibility of HIV and other sexually transmitted diseases spreading due to many sexual partners [7].

Intimate partner violence is a problem for global public health, particularly in low and middle-income countries [8]. Intimate partner violence can happen to women at any age but it is common among reproductive-age [9]. One in three women worldwide has at some point in their lives encountered one form of intimate partner violence [10]. According to a recently released multi-country study from 28 European Union, the prevalence of intimate partner violence was 26.1% [11]. Sub-Saharan Africa has a disproportionately high percentage of women experiencing intimate partner violence with an overall rate of 36% compared to the global average of 30% [7]. Despite the dearth of research on intimate partner violence in Africa, what is known indicates that of women who have ever been in a relationship, 37.14% of them had suffered IPV at some point in their lives as this study was conducted in 2023 [12]. The prevalence of intimate partner violence was 28.74% in Ethiopia conducted in 2022 [13]. Study conducted in Kenya in 2022 and 2021 is one of the African nations with a high rate of intimate partner violence, where estimates of the lifetime prevalence range from 20 to 78% depending on the type of population (among pregnant women, reproductive-age women, and married women) [14, 15]. Another study conducted in Kenya/2013 and Nigeria/2018 indicated 37% and 23.6% respectively [16, 17]. The burden of any form of IPV among women in Kenya conducted in 2022 revealed 60.30% [18].

Additionally, studies conducted among reproductive-age women provided variables that enhance the risk of IPV. The factors that were associated with IPV from different literature include; the youngest age, no occupation/job, low educational level, women’s decision-making freedom, husband alcohol use, and having several children [19, 20]. The other factors associated with IPV were residence, occupation of respondent and husband, partner education level, age difference between spouse, marital duration, women’s attitude towards partner beat, and male dominant behavior over female [21–23]. The other factors which were associated with intimate partner violence include; food problems, sleep issues, inactivity, and low self-esteem [24].

The Kenyan government has acknowledged that the main reasons for intimate partner violence are discrimination and gender inequality [15]. Although there is a large global frequency of intimate partner violence among pregnant women, studies conducted among reproductive-age women also show a high burden of IPV [25]. Even though studies have been conducted among reproductive-age women in different countries, there are limitations of studies particularly in Kenya. Therefore, this study is aimed at determining the associated factors of intimate partner violence among reproductive-age women at the individual and community level from the recent DHS 2022 data of Kenya.

## Method

### Study design and setting

Community-based cross-sectional study design was employed using the Demographic Health Survey of 2022 data in Kenya. Demographic Health Survey data was collected in 2022 in Kenya. The DHS sample size was computed with a total of 42,300 households, with 25 households selected per cluster, which resulted in 1,692 clusters spread across the country. From the total of households’ domestic violence was assessed among 30,456 households 20,304 were assessed for short questionnaires and 10,152 long questionnaires for women from 15 to 49 years old. The number of women eligible (33, 879), the number of eligible women interviewed (32,156), and 16,926 women aged 15–49 (unweighted) eligible for the module were interviewed successfully with World Health Organization’s guidelines (WHO) 2001. The Kenya Household Health Survey framework selected 1,692 clusters using the equal probability selection method (EPSEM). The KDHS datasets included variables on men, women, children, births, and households. The Individual Record dataset (IR file) was the data that have been extracted from this survey. Participants, who were reproductive-age women ages 15 to 49, from the Kenyan community served as the source populations. The final weighted sample size of this secondary data analysis was 14,612 reproductive-age women from the Kenya DHS. Detailed information on the data is available on the official link http://www.dhsprogram.com/.

### Variables of the study

#### Dependent variable

Intimate partner violence includes physical, sexual, and emotional: that were the dependent variables of the study which were measured by self-reported questioners of modified Conflict Tactic Scales of Straus [26]. The IPV was measured by the following nine questions and having at least one form of violence was considered as having intimate partner violence. The outcome variable was dichotomized based on participants who have IPV were recoded as 1 and don’t have IPV as 0.

#### Physical violence

Ever been kicked or dragged by your husband?

Ever been strangled or burned by a husband?

Ever been threatened with a knife, gun, or another weapon?

#### Sexual violence

Ever been physically forced to have unwanted sex by your husband?

Ever been forced to do other sexual acts by your husband?

Ever been forced to perform sexual acts respondent didn’t want to?

#### Emotional violence

Ever been humiliated by your husband?

Ever been threatened with harm by your husband?

Ever been insulted or made to feel bad by your husband?

#### Economic violence

Restrict, exploit, or sabotage your ability to acquire access, or maintain economic resources.

#### Independent variables

Independent variables were extracted from the Kenya DHS 2022 data including household variables, reproductive-related variables, and wealth index. The extracted independent variables for this study included; age, sex of the household head, number of alive under five children, distance from health facility, current marital status, religion, ethnicity, residence, employment status of respondent and husband, education level of respondent and husband, the age difference between spouse (subtract the age difference between spouse), and husband/partner ever alcohol use were used as individual-level variables. Community-level variables used for this study included place of residence (urban and rural), educational level (low and high), wealth index (low and high), and media exposure (low and high) calculated by adding listening to the radio, watching TV, and reading newspapers. Community-level variables are calculated based on the number of clusters included in the study. The community level variable and the clustered are run together in the Stata. The value of those two variables’ proportion was calculated in an Excel spreadsheet to get the community-level variable, then it was dichotomized based on the normality. Finally, mean and median were used for normal and skewed distribution respectively. The histogram was used to examine the distribution of the proportion values that were calculated for every community-level variable.

### Data analysis and management

Data extractions, coding, cleaning, and, analysis were conducted by using Stata version 14 software. Frequency and percentage were among the descriptive statistics that were completed in a table and text. Using sample weight with cluster, the analysis’s nonproportionate allocation and representativeness of the sample were carried out. An analysis that was mixed multilevel was carried out to preserve the collected data’s hierarchical structure.

Multilevel bivariable logistic regression analysis was conducted to determine the associated variables to be entered into multivariable analysis with a p-value of less than 0.25 [27]. Multilevel multivariable logistic regression analysis was used to determine the statistically significantly associated variables with a p-value of less than 0.05 and Adjusted Odd Ratio (AOR) with a 95% Confidence Interval (CI) was calculated.

For the multivariable multilevel logistic regression analysis, four model analyses were conducted. The initial model, also known as the null model, was run without the use of any explanatory variables. Only the individual-level variables were fitted in the second model; community-level variables were included in the third model; and both individual and community-level variables were fitted in the fourth model. Deviance and the Akaike Information Criterion (AIC) were employed to compare and assess the fitness of the models; the model with the lowest score was deemed to be the best fit. Additionally, the Intra-Class Correlation (ICC) was used to measure the degree of heterogeneity of women who have intimate partner violence across the clusters (the proportion of the overall observed individual variance in intimate partner violence that can be attributed to differences between clusters). Median Odds Ratio (MOR) was used to quantify the variation of intimate partner violence across clusters [28]. The degree of homogeneity of the assessment of intimate partner violence, and the measurement of odd ratio scale variation of intimate partner violence in the cluster, were carried out respectively. Finally, the AOR with 95% CI was calculated and variables statistically significantly associated with intimate partner violence were determined. We have conducted Hosmer and Lemeshow test (68.5) to determine the model fitness.

## Results

### Descriptive characteristics of respondents

A total of 15,127 reproductive-age women aged 15 to 49 were included in this secondary data analysis. Of the women, 64.18% were male-headed households, and 33.75% of the women were Protestant religion followers. Of the women,64.32% of the women were currently married and 81.97% of the women had been exposed to the media. Of the women, 90.53% had health service distance problems. Of the women, 73.02% had partner/husband alcohol users (Table 1).

**Table 1:** Descriptive characteristics of the women secondary data analysis

Variables	Category	Weighted frequency	Percentage
**Sex of the household head**	Male Female	9,309 5,203	63.70 35.60
**Age**	15–24 25–34 35–39 40–49	3,612 6,014 2,100 2,894	24.71 41.15 14.37 19.80
**Spouse age difference**	< 5 years *≥* 5 years	8,531 6,081	58.38 41.61
**Religion**	Catholic Protestant Evangelical Others*	2,605 5,006 3,200 3,801	17.82 34.25 21.89 26.01
**Women education**	No educations Primary Secondary and above	853 5,461 8,298	5.83 37.37 56.78
**Ethnicity**	Kalenjin Kamba Kikuyu Luhya Luo Meru Somali and Kisii Others ethnicity **	3,014 1,149 2,150 1,827 2,141 1,083 1,765 1,483	20.62 7.86 14.71 12.50 14.65 7.41 12.07 10.14
**Number of children**	No child One to two Three and more	5,316 8,452 844	36.38 57.84 5.77
**Wealth index**	Poorest Poorer Middle Richer Richest	2,285 2,556 2,755 3,367 3,649	15.63 17.49 18.85 23.04 24.97
**Distance from health facility**	Problem No problem	1,133 13,479	7.75 92.24
**Marital status**	Never in union Married Widowed/separated	3,151 9,492 1,969	21.56 64.96 13.47
**Partner/household education**	No educations Primary Secondary and above	1,023 4,102 9,487	7.00 28.07 64.92
**Partner/husband alcohol use**	No Yes	10,731 3,881	73.43 26.56
**Media exposure**	No Yes	2,312 12,200	15.82 83.49
**Women employment status**	Not working Working	4,933 9,679	38.90 61.10
**Paternal/husband employment status**	Not working Working	5,407 9,205	37.00 62.99
Community-level variables			
**Media exposure**	Low High	132 1,559	7.81 92.19
**Education**	Low High	1,625 66	96.10 3.90
**Wealth index**	Low High	864 827	51.09 48.91
**Residence**	Urban Rural	5,834 9,293	38.57 61.43

Legend

*Other religions include (Orthodox, Hindu, atheist, and traditionist)

**Other ethnicity (Embu, kisii, maasai, mijikenda/Swahili, taita/taveta)

### Prevalence of intimate partner violence

The overall prevalence of intimate partner violence was 41.1% with a 95% CI (40.07%, 42.60%). Of this prevalence 29.9% had physical violence, 27.8% had emotional violence, 10.3% had sexual violence and 10.7% had economic violence respectively (Table 2).

**Table 2:** The prevalence of each form of violence and overall intimate partner violence

Form of Violence	Frequency	Percentage
Emotional violence	4,369	29.9
Physical violence	4,062	27.8
Sexual violence	1,505	10.3
Economical violence	1,563	10.7
Any form of Physical or Emotional or Sexual or Economic violence	6,006	41.1

### Model fitness and statistical analysis

The ICC of the null model (model one) was 6.17% variations of the respondents related to the intimate partner violence attributed to the cluster. The null model’s MOR of intimate partner violence was 1.6 demonstrating that there was diversity amongst the clusters. If a single participant was randomly selected from each of the two clusters, the odds of that person having intimate partner violence were 1.6 times higher in the cluster with a higher risk of this violence than in the cluster with a lower risk. The best-fitted model for this study was model IV since it had the lowest value of deviance and AIC value. Model IV was conducted including both the individual-level variables and community variables. Therefore, model IV has incorporated the variables employed under model II and model III (Table 3).

**Table 3:** Multilevel multivariable logistic regression of Kenya Demographic and Health Survey data analysis

Variable	Category	Null model/ Model I	Model II	Model III	Model IV
**Sex of household head**	Male Female		1.04(0.95, 1.14) 1		**1.36(1.02, 1.81)** 1
**Wealth status**	Poorest Poorer Medium Richer Richest		1.75(1.48, 2,06) 1.69(1.46, 1.96) 1.51(1.32, 1.74) 1.37(1.21, 1.56) 1		**1.86(1.08, 3.21)** **1.78(1.04, 3.02)** **1.98(1.26, 3.08)** 1.31(0.90, 1.90) 1
**Distance from health facility**	Problem No problem		1.21(1.06, 1.37) 1		1.23(0.89, 1.70) 1
**Partner/husband education**	No education Primary Secondary & above		1.18(0.98, 1.41) 1.14(1.03, 1.26) 1		1.28(0.81, 2.03) 0.77(0.57, 1.04) 1
**Partner/husband alcohol use**	Yes No		4.10(3.76, 4.47) 1		**4.23(3.16,5 0.56**) 1
**Media exposure**	No Yes		1.25(1.11, 1.41) 1		1.02(0.76,1.37) 1
**Age**	15–24 25–34 35–39 40–49		1.06(0.96, 1.18) 1.09(0.96, 0.25) 1.18(0.96, 0.35) 1		0.83(0.61, 1.12) 0.88(0.60, 1.28) 0.93(0.63, 1.37) 1
**Women employment status**	Not working Working		1.33(1.22, 1.41) 1		1.18(0.92, 1.51) 1
**Partner employment status**	Not working Working		1.13(1.01, 1.27) 1		0.78(0.55, 1.10) 1
**Number of children**	No child One to two Three and more		1 1.04(0.96, 1.14) 0.96(0.80, 1.15)		1 1.28(0.99, 1.66) 1.34(0.85, 1.13)
**Marital status**	Not in union Married Separated/widowed		1 2.23(1.88, 2.66) 3.60(3.09,4.20)		1 0.95(0.55, 1.65) **2.02(1.24, 3.28)**
**Women education**	Ne education Primary Secondary & above		1.08(0.90, 1.29) 1.35(1.23, 1.48) 1		**1.56(1.02, 3.38)** **1.51(1.13, 2.02)** 1
**Community level variables**					
**Residence**	Urban Rural			1 1.24(0.95, 1.60)	1 0.83(0.58 ,1.17)
**Media exposure**	High Low			1 0.31(0.75, 2.05)	1 1.46(0.86, 1.50)
**Education**	High Low			1 0.34(0.51, 1.95)	1 1.12(0.55, 2.26)
**wealth status**	High Low			1 0.10(0.18, 0.60)	1 1.02(0.81, 1.27)
**Likelihood ratio**		-10023.49	-8953.1333	-1101.8945	-1016.2628
**ICC**		0.1110699	0.0907771	0.075085	0.061777
**Deviance**		20046.98	17906.267	2203.7889	2032.5257
**AIC**		2215.789	2086.526	17955.27	17952.27
**BIC**		2248.387	2233.219	18127.62	18127.62
**MOR**		1.60			

Legend

*ICC: Intra-Class Correlation

*MOR: Median Odds Ratio

*AIC: Akaike Information Criterion

*BIC: Bayesian Information Criteria

### Associated factors of intimate partner violence

In bivariable multilevel logistic regression analysis, the factors associated with intimate partner violence were age, respondent employment status and education, partner education, and employment status, number alive under five children, sex of the household head, current marital status, wealth status, distance from the health facility, partner alcohol use, and media exposure from the individual level variables and no variable is associated at the community level. In multivariable multilevel logistic regression analysis male-headed households, poorest and middle wealth status, partner alcohol use, separated/widowed marital status, and no and primary education of women were statistically significantly associated with intimate partner violence at the individual level variable. The odds of having intimate partner violence were 1.36 times higher among male-headed households than the other female-headed households [AOR = 1.36; 95%CI: (1.02, 1.81)]. The odds of experiencing intimate partner violence were 1.86 times, 1,78 times, and 1.98 times more having the poorest, poorer, and middle as compared to the richest participants {AOR = 1.86; 95%CI: (1.08, 3.21), AOR = 1.78; 95%: (1.04, 3.02), and AOR = 1.98; 95% CI: (1.26, 3.08)}. The odds of intimate partner violence among partner alcohol users were 4.23 times more than those who have a partner not used alcohol [AOR = 4.23; 95% CI: (3.16,5 0.56)]. Being separated/widowed were 2.02 times more likely to have intimate partner violence as compared to not in union [AOR = 2.02;95% CI: (1.24, 3.28)]. The odds of experiencing intimate partner violence among women who had been not educated were 1.56 and 1.51 times high as compared to having high educational status [AOR = 1.56; 95% CI: (1.02, 3.38)] and [AOR = 1.51; 95% CI: (1.13, 1.02)] (Table 3).

## Discussion

The overall prevalence of intimate partner violence from the recent Kenya DHS was 41.1% with a 95% CI (40.07%, 42.60%). The prevalence of intimate partner violence conducted in Kenya DHS a secondary data analysis was high. More than four out of ten women of reproductive-age had suffered from intimate partner violence in Kenya based on the DHS dataset. This finding is lower than other studies conducted in Tanzania 61% [29] and Uganda 56% [30].The discrepancy in this result could be the effect of different risk variables for IPV, for instance, there are a lot of variables associated in Uganda and Tanzania that were not at risk for this study [29, 30]. In other ways, this finding is also higher than other studies conducted in Ethiopia 34% [13] and Nigeria 23.6% [31].The other reason for the difference could be because of unreported violations or the issue that comes from the cultural background of Ethiopian women [13].

All of the variables associated with IPV were from the individual level and community-level variables were not associated in this study. Related to the factors associated with intimate partner violence were the male-headed households. This finding is similar to a study conducted in Nigeria [31]. Male dominance and their spouses’ attitudes toward their partners are the sources of conflict, which eventually results from intimate partner violence. Male-headed households always lack strong relationships and share a load of housework leading couples to feel not agreed at running their home tasks [31]. Male-headed household is associated because of women’s lack of decision-making authority which exposes them to intimate partner violence.

Another factor that was associated with intimate partner violence was the lower wealth status class. This finding is in concordance with other studies conducted in Ethiopia [32] and Nigeria [33]. This is because of previous contradictory findings from research done in low- and middle-income nations, in terms of the strength and direction of the wealth-related link as well as the evidence of statistical significance [32]. While some researchers have discovered an adverse relationship, others have identified a positive relationship [34]. The results of this study are consistent with other study conducted in Sub-Saharan Africa [33].

The other factor associated with intimate partner violence was partner alcohol use. This result is in line with other studies conducted in Ethiopia [35]. This association might be due to the effect of alcohol drunk can result in careless actions, such as impaired judgment and comprehension of social standards, which raises the risk of intimate partner violence [35]. Drinking can lead to domestic violence, which can exacerbate bad relationships with stress and lead to violations. Partner alcohol drinking has also been associated with several sexual partners, which can lead to a problem that might cause conflict [36]. This could be because drinking alcohol has a direct impact on how people think and behave. This mental distortion may lead to users acting aggressively in relationships and a rise in violent incidents [37].

Being separated/widowed marital status was another factor which was associated with intimate partner violence. This finding is in line with other studies conducted in Canada [38], Bangladesh [39], Nigeria [40], and Ethiopia [41]. The high prevalence of intimate partner violence might be the reason that leads to separated/widowed. The possible reason for the association could be the effect of women might be divorced/separated due to the effect of violation of their husband [40].

The low education status of reproductive-age women was the other factor associated with intimate partner violence. This is consistent with other studies conducted in Nepal [42, 43] and Ethiopia [13]. This could be because reproductive-age women without formal education might not have as much influence over their partners to resolve disagreements in the home [43]. This association could be because women with high literacy or education have better access to know about women’s rights, or it could be because they are less likely to accept partner violence than uneducated women [42]. One explanation could be that women who have higher levels of education have a higher chance of landing well-paying positions, rising through the ranks, and having a more equal authority dynamic in relationships [44]. Generally, women with higher education levels are less tolerant of forced sex, beatings, and chokeholds and their husbands’ mistreatment and power over them.

### Strengths and limitations

The utilization of a large sample size (15,127) from the national DHS dataset based in Kenya makes this analysis more generalizable to determine intimate partner violence. There is adequate power to determine the true influence of the independent variables when using data from a big countrywide survey. However, it has also limitations; like the cross-sectional nature of the data that cannot indicate the temporal relationship. The DHS data did not include the most vulnerable people who were on the streets, refugees, incarcerated /or other types who are out of household or institutionalized. The other limitation of this study is the social desirability bias since intimate partner violence leads to labeling their privet life conditions. The data-specific measurement tool of variables was not determined and the women beyond reproductive-age were not included because the study used secondary data analysis from DHS.

### Conclusion and recommendations

The prevalence of intimate partner violence was high. Educating women, reducing partner alcohol use, and improving the economic status of women, were crucial in mitigating the burden of intimate partner violence. The intimate partners are always supposed to respect the rights of women to reduce the burden of IPV. The Kenya policymakers are recommended to strengthen their work related to intimate partner violence. The advanced study design is recommended for future researchers to know the cause and effect of the association of factors with IPV.

## Data Availability

The datasets generated and/or analyzed during the current study are available in the measure of the DHS program repository, http://www.dhsprogram.com.

## Abbreviations

AIC: Akaike Information Criteria
AOR: Adjusted Odds Ratio
DHS: Demographic Health Survey
CI: Confidence Interval
ICC: Intra-Class Correlation
MOR: Median Odds Ratio
PCV: Proportional Change in Variance
WHO: World Health Organizations
